# Construction and validation of a comprehensive metabolism-associated prognostic model for predicting survival and immunotherapy benefits in ovarian cancer

**DOI:** 10.7150/jca.100796

**Published:** 2024-09-23

**Authors:** Wei Ye, Yuanyuan Fang, Zhaolian Wei

**Affiliations:** 1 Department of Obstetrics and Gynecology, The First Affiliated Hospital of Anhui Medical University, Hefei, Anhui 230022, China.; 2 Department of Obstetrics and Gynecology, The Second Affiliated Hospital of Anhui Medical University, Hefei, Anhui 230601, China.

**Keywords:** Ovarian cancer, Cancer metabolism, P2RY14, Prognosis, Immune landscape

## Abstract

**Background:** Ovarian cancer (OV) is a prevalent malignancy among gynecological tumors. Numerous metabolic pathways play a significant role in various human diseases, including malignant tumors. Our study aimed to develop a prognostic signature for OV based on a comprehensive set of metabolism-related genes (MRGs).

**Method:** To achieve this, a bioinformatics analysis was performed on the expression profiles of 51 MRGs. The OV individuals were subsequently categorized into two molecular clusters based on the expression levels of MRGs. Following this, differentially expressed genes (DEGs) were identified among these clusters. The DEGs aided in the classification of two gene clusters, with a total of 390 DEGs being identified between them. A prognostic signature, constructed using the DEGs, enabled the calculation of risk scores for OV patients.

**Results:** This study revealed that patients classified as low-risk demonstrated a more favorable prognosis, increased immune cell infiltration, and superior response to chemotherapy in comparison to high-risk patients. Four signature genes, GDF6, KIF26A, P2RY14, and ALDH1A2, were identified as significant contributors to the prognostic signature. The expression levels of these signature genes were different between OV and normal ovary tissues through in vitro experiments. Additionally, P2RY14 protein was found to potentially influence the growth of OV cell lines.

**Conclusion:** We have constructed a prognostic signature associated with MRGs that demonstrates exceptional efficacy in prognosis survival outcomes and therapeutic responses in patients diagnosed with OV. Downregulation of P2RY14 may contribute to an unfavorable prognosis in OV.

## Introduction

The incidence of ovarian cancer (OV) is among the highest of all gynecological tumors, and it is one of the most deadly^1^. The major risk factor for OV is the mutation of the BRCA1 gene, but other factors also includes increasing age, endometriosis, and so on^2^, ^3^.

Numerous trials have consistently encountered challenges in identifying appropriate biomarkers for early detection of OV among women in the general population^4^, resulting in a significant number of patients being diagnosed at an advanced stage^5^. Currently, the primary treatments for advanced OV patients include involve surgical intervention and neoadjuvant chemotherapy, and immunotherapy also has protential, but the efficacy is not ideal^6^, ^7^. Hence, it is necessary to build a more accurate prognostic signature model for OV.

In recent times, there has been a significant amount of research conducted to construct a prognostic signature for predicting the survival outcomes of individuals with tumors, such as malignant melanoma and colorectal cancer^8^, ^9^. During the process of constructing these signatures, the signature genes are also identified. Additionally, numerous studies have shown that changes in the metabolic pathways of cancer cells can impact the occurrence and progression of tumors^10^. Meanwhile, the impact of genetic mutations on tumor progression through the modulation of metabolic pathways, specifically HIF-1 and PDK1, already known as MRGs, has been extensively investigated^10^, ^11^. The main metabolic pathways include glucose, lipid, and amino acid metabolic processes^12^, ^13^.

Utilizing public databases of tumor samples to identify prognostic genes based on gene sets, which contain a group of genes with common characteristics, is a common and effective method^9^, ^14^, ^15^. These investigations have further incorporated data pertaining to tumor mutation burden (TMB) and tumor microenvironment (TME) to assess prognostic models^16^, ^17^. Nevertheless, as of now, no studies have successfully established a prognostic signature model for OV based on a substantial number of MRGs.

In this study, 51 prognostic-related MRGs (Supplementary Table 2) were selected from 2752 pan-MRGs (Supplementary Table 1) collected from the GO (Gene Ontology) database, and a prognostic model for predicting the prognosis of OV was established based on these genes and verified by some immune-related analysis. Meanwhile, we identified four signature genes: GDF6, KIF26A, P2RY14, and ALDH1A2. Interestingly, we also discovered that downregulating P2RY14 expression may suppress the migration and proliferation of OV cells, which indicates that low P2RY14 expression may correlate with poor prognosis in OV.

## Methods and materials

### Data collection

Transcription and clinical information of OV patients were retrieved from TCGA (https://portal.gdc.cancer.gov, TCGA-OV project) and GEO (https://www.ncbi.nlm.nih.gov/geo/, ID: GSE13876 and GSE23554) databases (Supplementary Table 2). Additionally, expression data of normal controls were downloaded from GTEx database. Expression matrices of 4 OV single cell datasets, including EMTAB8107, GSE130000, GSE151214, and GSE154600 were obtained from TISCH2 website (http://tisch.comp-genomics.org/). Fragments per kilobase million (FPKM) data from TCGA-OV dataset was transformed into transcription per million (TPM) format using R studio software, and expression data was further log2 transformed using “sva” R package.

### Genetic and transcriptional alterations of MRGs in OV

2752 MRGs were obtained from Gene Ontology (GO) database (http://geneontology.org/), identified DEGs in normal tissues and OV using the TCGA-GTEx dataset, and screened out 51 prognosis-related mrg in the TCGA-OV dataset with a p < 0.01 threshold using univariate Cox regression analysis. Somatic mutation data of 51 MRGs were analyzed using “maftools” R package and the results were presented in the waterfall plot. The frequency and genomic locations of copy number variations (CNVs) in 51 MRGs across chromosomes were scrutinized and delineated. Differential expression patterns between healthy controls and OV tissues were assessed utilizing the Wilcoxon test integrated within the limma package. Additionally, the prognostic significance and correlation of MRGs were appraised through a univariate Cox regression and Pearson analyses.

### Identification of two MRGs molecular clusters

Consensus clustering was conducted to delineate molecular clusters based on the expression profiles of 51 MRGs via the “ConsensusClusterPlu” R package. By incrementing the clustering parameter k, the categorization with maximal intragroup cohesion and minimal intergroup divergence was determined. Subsequently, Principal Component Analysis (PCA) was employed to discriminate between the identified molecular clusters. GO and Kyoto Encyclopedia of Genes and Genomes (KEGG) analyses were done to conduct an in-depth study of MRG-related biological functions and pathways utilizing xiantao online tool (https://www.xiantaozi.com). The Kaplan-Meier method was applied to assess disparities in survival duration across MRG clusters, with comparison facilitated through the log-rank test and R packages dedicated to survival analysis, such as “survival” and “survminer”. Clinical variables within MRG clusters were juxtaposed, and Differentially Expressed Genes (DEGs) were identified between clusters based on criteria including |log fold-change| > 0.585 and p < 0.05. Immunological cell infiltration and pathways associated with immune responses within MRG clusters were elucidated using Gene Set Variation Analysis (GSVA) and Single-Sample Gene Set Enrichment Analysis (ssGSEA) via the “gsva” R package. The expression levels of three prominent immune checkpoint genes across MRG clusters were assessed using the Wilcoxon signed-rank test and visualized using violin plots.

### Classifying patients into gene clusters according to DEGs between MRG clusters

To further identify genes for constructing the prognostic signature, expression of DEGs between MRG clusters was utilized to classify OV patients into geneclusters using the “ConsensusClusterPlus” R package. Clinical characteristics and MRGs expression across the gene clusters were evaluated using heatmaps and boxplots via the Wilcoxon signed-rank test. Survival times among the gene clusters were assessed using the Kaplan-Meier method, with comparisons made via the log-rank test. Additionally, the expression levels of PD-1, PD-L1, and CTLA-4 within the three gene clusters were analyzed.

### Construction and verification of the MRGs-related prognostic signature

Differentially expressed genes (DEGs) were identified across the three gene clusters. Utilizing these DEGs, least absolute shrinkage and selection operator (LASSO) regression and multivariate Cox regression analyses were conducted to identify genes for constructing a predictive signature, employing the survival, survminer, and glmnet R packages. The risk score was calculated using signature genes expression and relative regression coefficient values. Based on the calculated risk scores, individuals diagnosed with OV were stratified into high-risk and low-risk groups. The study analyzed the associations between risk score, survival time, and survival status. Additionally, univariate and multivariate Cox regression analyses were conducted to identify significant predictive variables in OV patients. These analyses used the risk score alongside other pertinent clinical features. Furthermore, a nomogram model was constructed based on the results of Cox regression, and the efficacy of the model was evaluated using calibration plots. The efficacy of the prognostic signature in predicting OV patient survival was validated in the training cohort and 2 independent cohorts (GSE13876 and GSE23554) using Kaplan-Meier survival analysis and receiver operating characteristic (ROC) curve methods.

### Exploring the immune landscape of OV patients in low- and high-risk groups

The CIBERSORT algorithm was applied to quantify the infiltration of immunological cells in TCGA-OV patients. The correlation between risk scores and immunological cell abundance was then assessed using the Spearman correlation technique Furthermore, the relationship between immunological cell profiles and 4 signature genes was investigated. Tumor Microenvironment (TME) scores, including stromal, immune, and ESTIMATE scores among high- and low-risk cohorts were evaluated utilizing the Wilcoxon signed-rank test, with graphical representation facilitated by violin plots. Additionally, the Cancer Stem Cell (CSC) index within both risk groups were examined using the Wilcoxon signed-rank test in conjunction with the Spearman correlation analysis.

### IC50 of therapeutic drugs in low- and high-risk groups

The IC50 (half-maximal inhibitory concentration) is a parameter that quantifies the concentration of a therapeutic agent required to inhibit 50% of cancer cell viability. To assess the differential sensitivity of high-risk versus low-risk groups to various therapeutic drugs, the IC50 values were analyzed using the “pRRophetic” R package and the Wilcoxon signed-rank test.

### Expression of 4 signature genes in different cell types using single cell sequencing

Single-cell expression matrices from the EMTAB8107, GSE130000, GSE151214, and GSE154600 datasets were obtained from the TISCH2 website (http://tisch.comp-genomics.org/). Cell type annotations were assigned based on the expression profiles of specific marker genes, employing the Monaco Immune Database within the Celldex package. Following this, the expression patterns of 4 signature genes were visualized across different cell types for further analysis. Furthermore, expression levels, prognostic values, and correlation with immune checkpoints of 4 signature genes in OV were analyzed using the xiantao online tool.

### The comparison of expression levels of the four signature genes in OV and control group

A comparison was conducted between the expressed levels of the four signature genes in OV samples and normal controls.

#### Cell culture

The OV cell line (A2780 and OVCA433) and the normal ovarian cell line (IOSE) were acquired from the American Typical Culture Collection (ATCC). Additionally, IOSE, OVCA433 and A2780 cell lines were incubated in DMEM (HyClone) supplemented with 10% fetal calf serum (Lonsera) and 1% double antibody (streptomycin and penicillin) at a temperature of 37°C and 5% CO2.

#### qPCR

The mRNA expression of the four signature genes was compared between the OV cell line (A2780) and the normal ovarian cell line (IOSE) using qPCR. The primer sequence can be found in supplementary table S4, and the relative mRNA expression levels of the genes were calculated using the 2^-ΔΔCt^ method.

#### OV Tissue Microarray and IHC

OV and normal ovarian tissue microarray (ZL-OVA961) were bought from ShangHai Zhuoli Biotech Company (China). Then, IHC staining was utilized to measure the protein expression of GDF6 and P2RY14 using anti-GDF6 (BIOSS, China) and anti-P2RY14 antibodies (BIOSS, China).

#### Exploration of the function of P2RY14 in OV

The correlation between the expression levels of all eight immune checkpoint genes and those of P2RY14 were explored. Meanwhile, we also explored the impact of P2RY14 expression on ovarian carcinoma cell proliferation and migration capacity.

#### Cell transfection

The transfection of small interfering RNA (siRNA) was conducted utilizing Lipofectamine 3000 (Invitrogen, Shanghai, China) in accordance with the manufacturer's instructions. After a 48-hour incubation period, the cells were rinsed and utilized for subsequent experiments. The sequences were as follows: 5'-CAGAUCAUUCCUGUGCUGUACUGUA-3' for P2RY14-specific siRNA1, 5'-CCGUGCUCUUCUACGUCAACAUGUA-3' for P2RY14-specific siRNA2.

#### WB

Protein extraction from IOSE, OVCA433 and A2780 cells was performed by utilizing RIPA buffer (Beyotime, China) containing protease inhibitor and phosphatase inhibitor. The Western blot technique was carried out according to a preexisting procedure. In this research, the main antibodies used were anti-Tubulin from Proteintech in China and anti-P2RY14 from BIOSS in China.

#### CCK8 methods

A2780 and OVCA433 cells were incubated into 6-well plates with a density of 200,000 cells per well and subsequently transfected with either NC-siRNA or siRNAs targeting P2RY14 (specifically siRNA1 or siRNA2). After 48 hours of transfection, 1,500 cells were seeded into 96-well plates. Subsequently, the cells were cultured with either NC-siRNA or siRNAs targeting P2RY14 (specifically siRNA1 or siRNA2) for 0, 24, 48, or 72 hours. Subsequently, the above cells were subjected to treatment with the CCK8 solution (Beyotime, Shanghai, China) for 1.5 hours. Cell viability was assessed by measuring the optical density (OD) value at 450 nm.

#### Colony formation assay

To determine the impact of P2RY14 expression on the proliferation of human OV cells, the aforementioned transfected A2780 and OVCA433 cells (2000/well) were introduced into 6-well plates. Following a ten-day incubation period, the colonies were counted.

#### Transwell experiment

The migration experiment utilized Transwell chambers (Corning, NY, USA). The above mentioned A2780 and OVCA433 cells, which were transfected at a concentration of 3 × 104, were seeded in 200 μl of serum-free DMEM and subsequently introduced into the upper chamber. The lower chambers were added with a DMEM containing 10% fetal bovine serum. Following an incubation period of 36 hours, the inner chambers were cleaned and the cells on the opposite side of the membrane were fixed using a 4% formaldehyde solution. Subsequently, staining with crystal violet was performed, and the samples were examined under a microscope.

## Results

### The genetic and transcriptional changes of MRGs in OV

**Figure 1:**
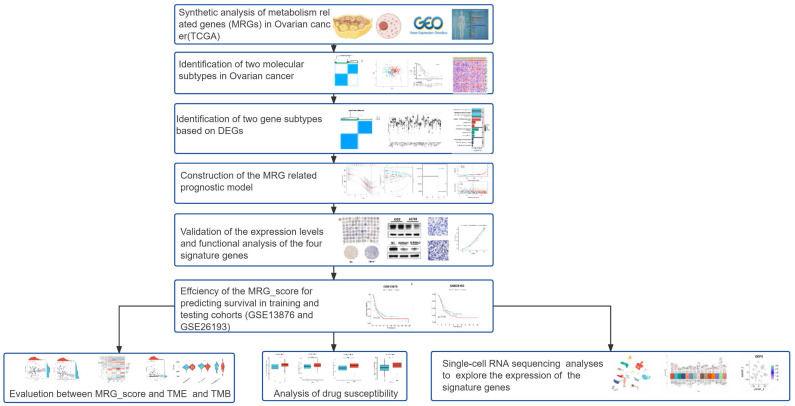
Work flow of this research.

**Figure 2:**
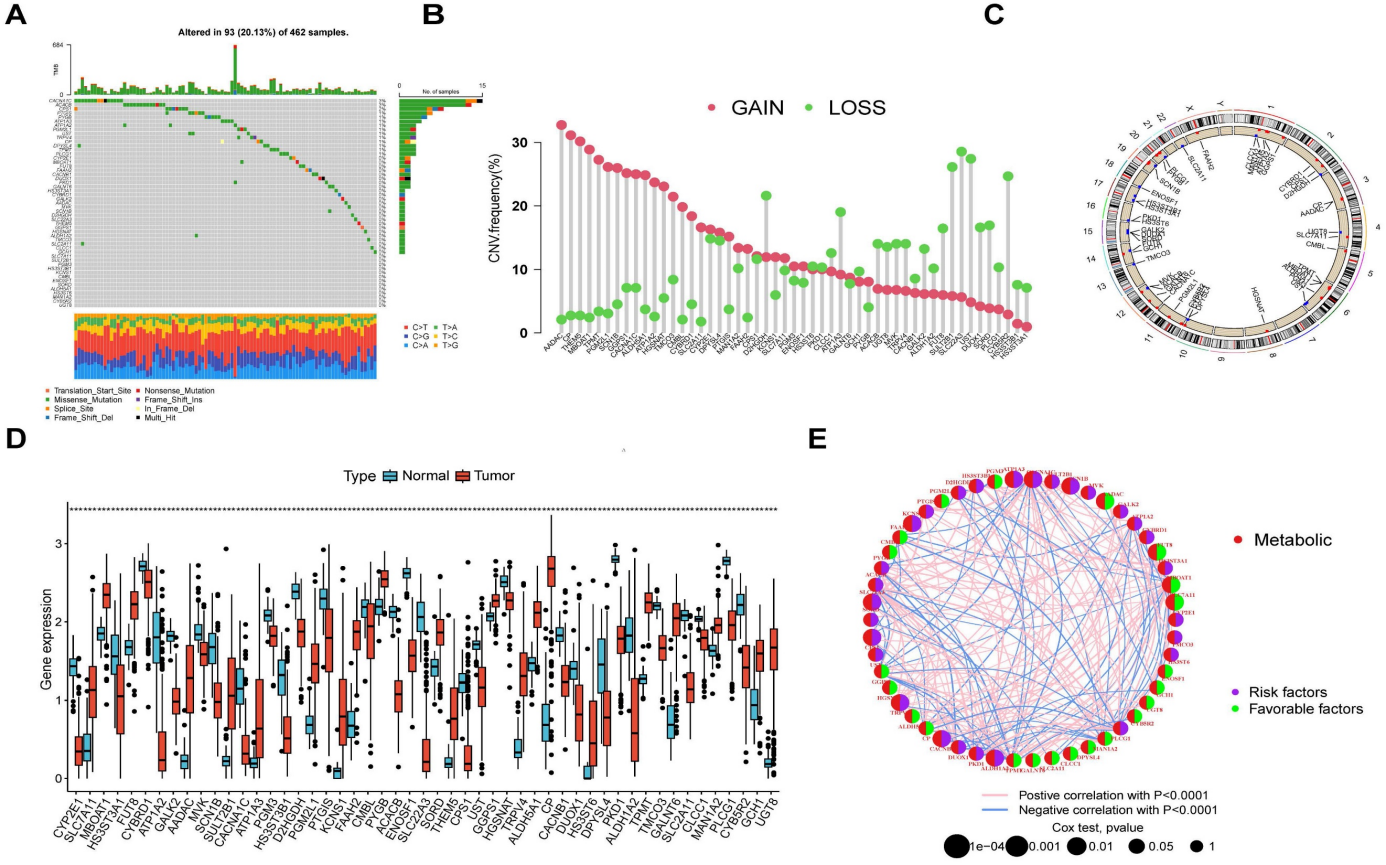
Presents the results of a genetic analysis conducted on MRGs in OV. (A) The somatic mutation rate among the 51 MRGs in OV individuals. (B) The CNV alterations among 51 MRGs. (C) Locations of CNV changes in MRGs on 23 chromosomes. (D) The difference of expressed levels of MRGs in tumor patients and healthy controls. (E) The present study examines the interactions between MRGs in OV, with blue and red lines indicating positive and negative correlations, respectively. *p < 0.05; **p < 0.01; ***p < 0.001.

A flow diagram of this research is displayed in Figure 1. To explore the genetic alteration of MRGs in OV patients, we analyzed the mutation data of MRGs and found 93 (20.13%) somatic mutations in 462 OV patients (Figure 2A). we also found many genes showed CNV increase such as AADAC, CP, THEM5, TPMT and PGM2L1, while CYB5R2, UST, SLC22A3, SULT2B1 and D2HGDH showed CNV loss (Figure 2B). Furthermore, the location of the CNV changes on chromosomes was illustrated in Figure 2C. In addition, a comparison was made between the expression levels of MRGs in OV individuals and normal healthy controls. A significant number of MRGs exhibited differential expression between the OV samples and controls (Figure 2D). A network demonstrated the prognostic significance of MRGs interactions (Figure 2E). These results suggested that there are many genetic alterations of MRGs in OV patients.

### Identification of two MRG molecular clusters in OV

**Figure 3:**
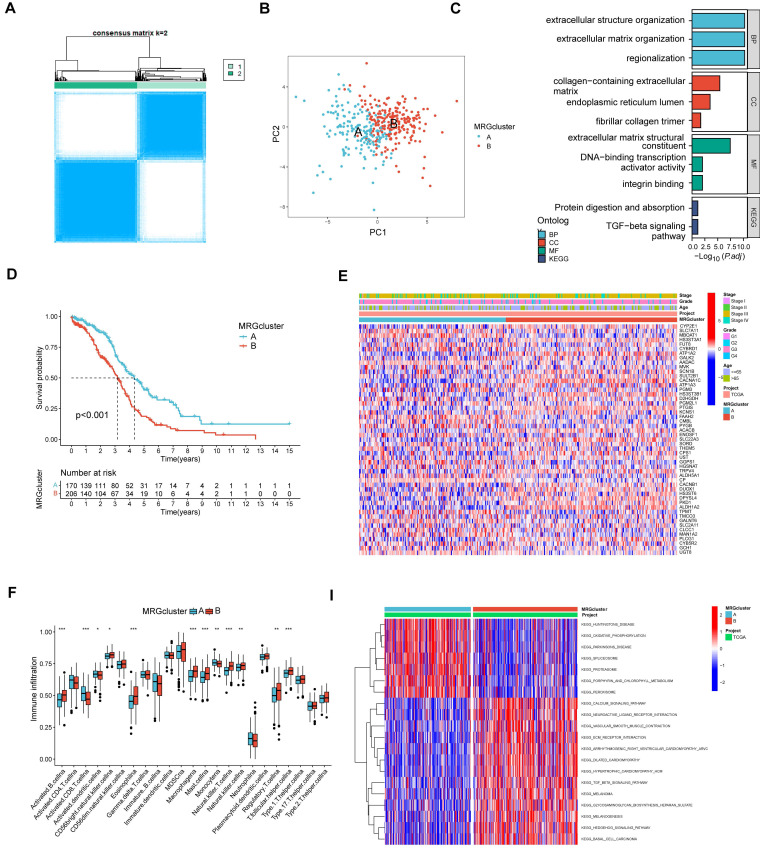
The comparison between the two molecular clusters. (A) Identifying two MRG molecular clusters utilizing consensus clustering analysis. (B) Principal Component Analysis effectively illustrated the differentiation between the two molecular clusters. (C) GO and KEGG analysis of the MRGs. (D) The Kaplan-Meier curve proved a statistically significant disparity in survival time between the two clusters. (E) Heatmaps demonstrated the clinical features and MRGs expression of the two molecular clusters in OV patients. (F) The differences in immune cell infiltration between two molecular clusters were investigated using ssGSEA. (I) GSVA revealed the enriched pathways between the two molecular clusters. *p < 0.05; **p < 0.01; ***p < 0.001.

Using MRGs expression levels as a basis, we employed a consensus clustering method to classify OV patients into two distinct molecular clusters, designated as clusters A and B (Figure 3A). Additionally, PCA analysis confirmed the distinct separation between these two molecular clusters (Figure 3B). The analysis of functional enrichment for these MRGs revealed that the aforementioned MRGs are primarily enriched in biological process (BP) related to the organization of extracellular structures, cellular component (CC) involving collagen-containing extracellular matrix, molecular function (MF) associated with extracellular matrix structural constituent, and protein digestion and absorption pathways (Figure 3C). The K-M curve indicated the molecular cluster A had a better survival than cluster B (Figure 3D). Furthermore, a heatmap showed the MRGs expression levels and clinical characteristics of the two clusters (Figure 3E). Meanwhile, it was also found there were significant differences in many immune cell infiltrations in the two clusters by using ssGSEA methods (Figure 3F). The differentially enriched pathways between the two MRG clusters are illustrated in Figure 3G. Additionally, we observed distinct clinical features and survival outcomes between the two clusters of OV patients.

### Identification of two gene clusters in OV

**Figure 4:**
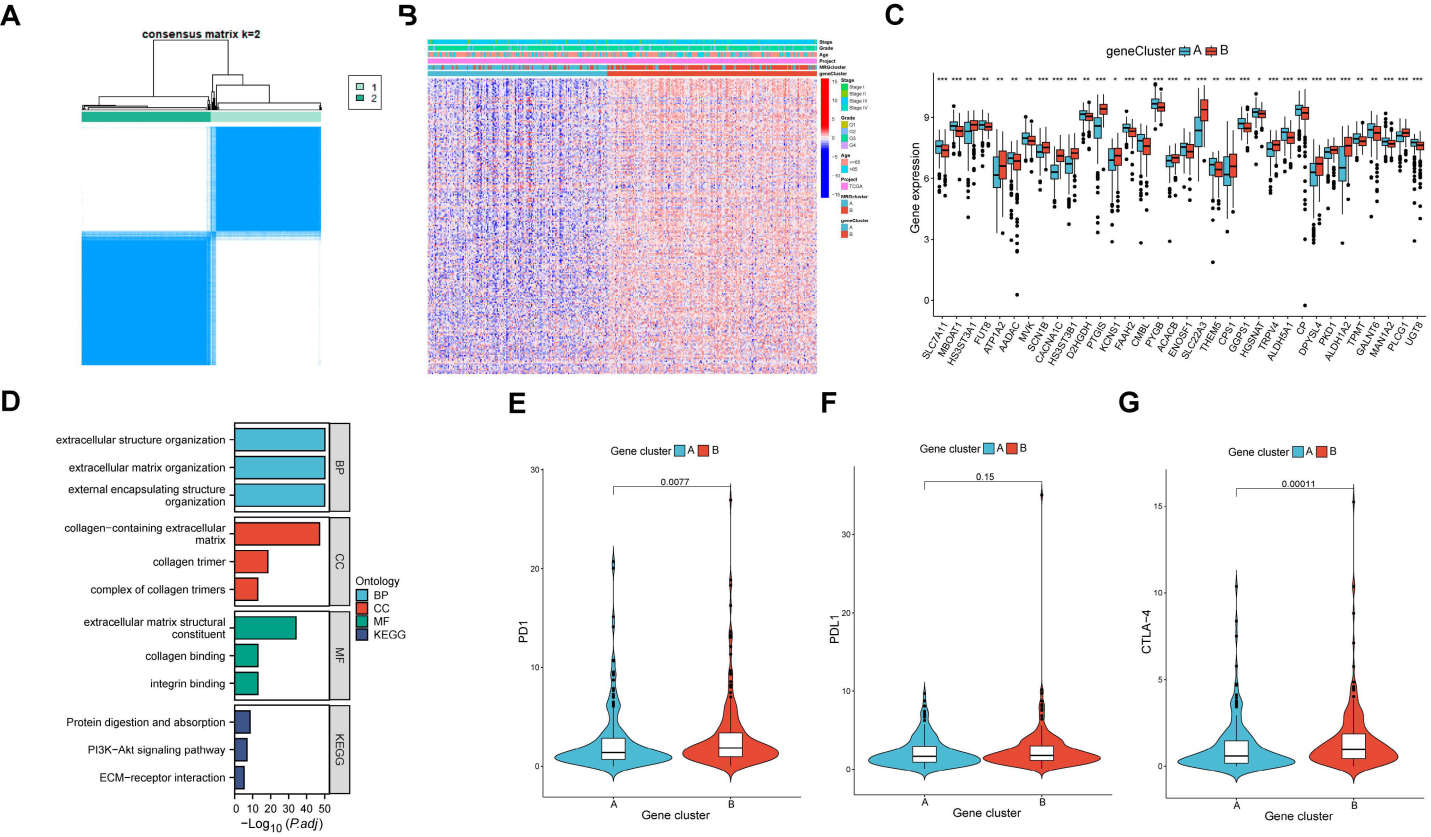
The comparison between the two gene clusters. (A) Consensus clustering analysis was utilized to identify two gene molecular clusters based on the DEGs between the two MRG clusters. (B) Heatmaps showed the clinical features and MRGs expression of the two gene clusters in OV individuals. (C) Expression of 51 prognostic related MRGs between the two gene clusters (D) GO and KEGG analysis of the DEGs. (E-G) Different expressed PD-1, PD-L1, and CTLA-4 among the two gene clusters.

DEGs were identified between the two molecular clusters, followed by the identification of two gene clusters of OV samples based on the DEGs (Figure 4A). Furthermore, a total of 390 DEGs were identified between the two clusters. Similarly, significant differences were observed in the expression levels of MRGs and clinical characteristics between the two gene clusters (Figure 4B). There were significant differences in the 51 prognostic related MRGs between the two gene clusters (Figure 4C). By conducting functional enrichment analysis on the DEGs, it was observed that these genes primarily exhibited enrichment in biological processes related to extracellular structure organization, cellular components associated with collagen-containing extracellular matrix, molecular functions involving extracellular matrix structural constituents, and pathways related to protein digestion and absorption (Figure 4D). Notably, the expressed levels of three prominent immune checkpoint genes, namely PD1, PDL1, and CTLA-4, exhibited variations between the two gene clusters (Figure 4E-G).

### Construction and validation of the MRGs-related prognostic signature

**Figure 5:**
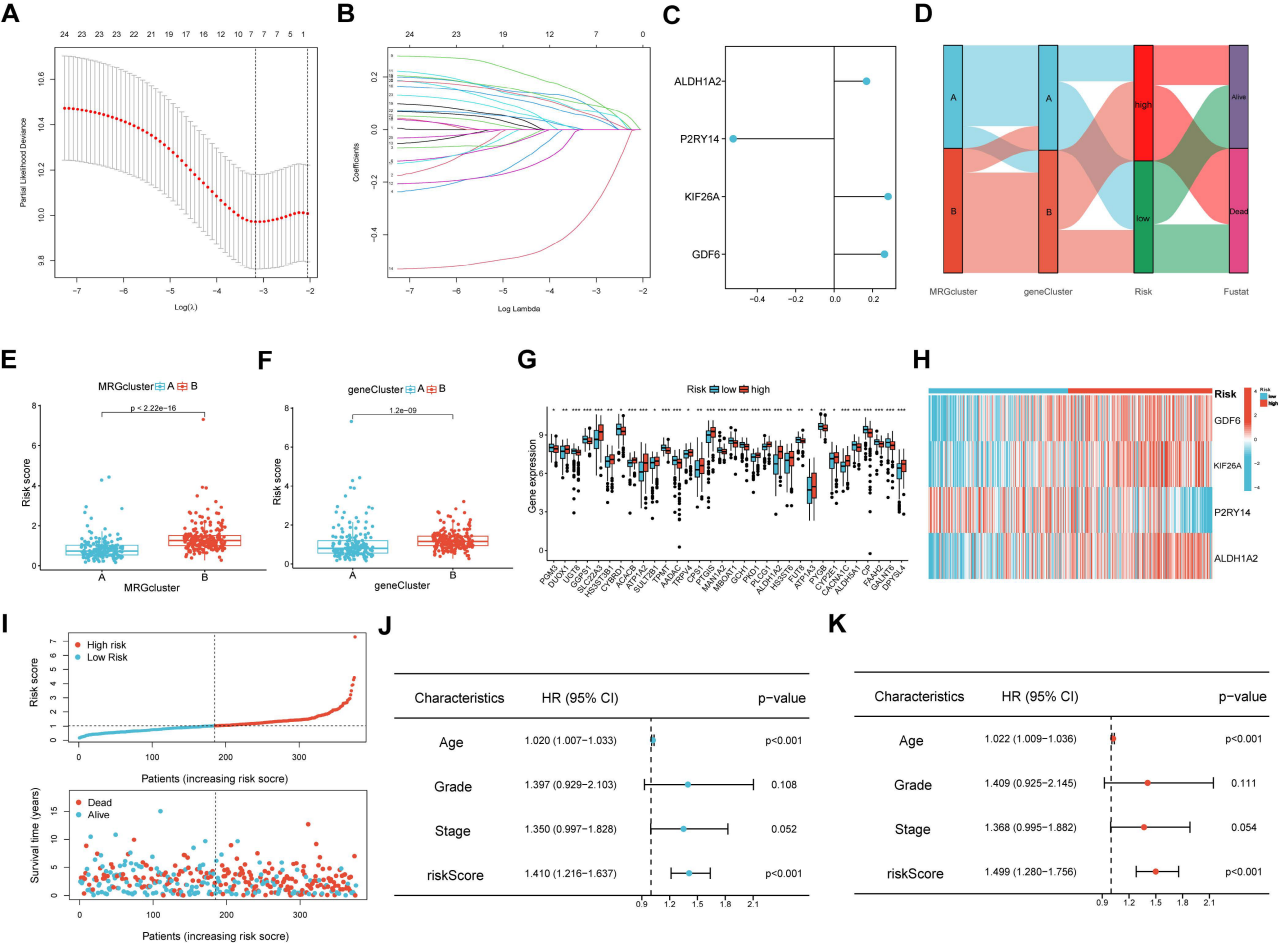
Development of the prognostic signature. (A, B) Partial likelihood deviance and the LASSO regression analysis on the prognostic signature genes. (C) The coefficient values of the multivariate Cox regression. (D) Sankey diagram exhibited the two molecular clusters, two gene clusters and two risk groups. (E, F) Differences in risk score among the two molecular clusters and two gene clusters. (G) Differences in expression levels of MRGs in the two risk groups. (H)The heatmap showed the expression levels of the four signature genes among the two risk groups. (I) Risk score and survival status of OV individuals. (J-K) The risk parameters of single and multiple factors Cox regression analyses in OV individuals.

**Figure 6:**
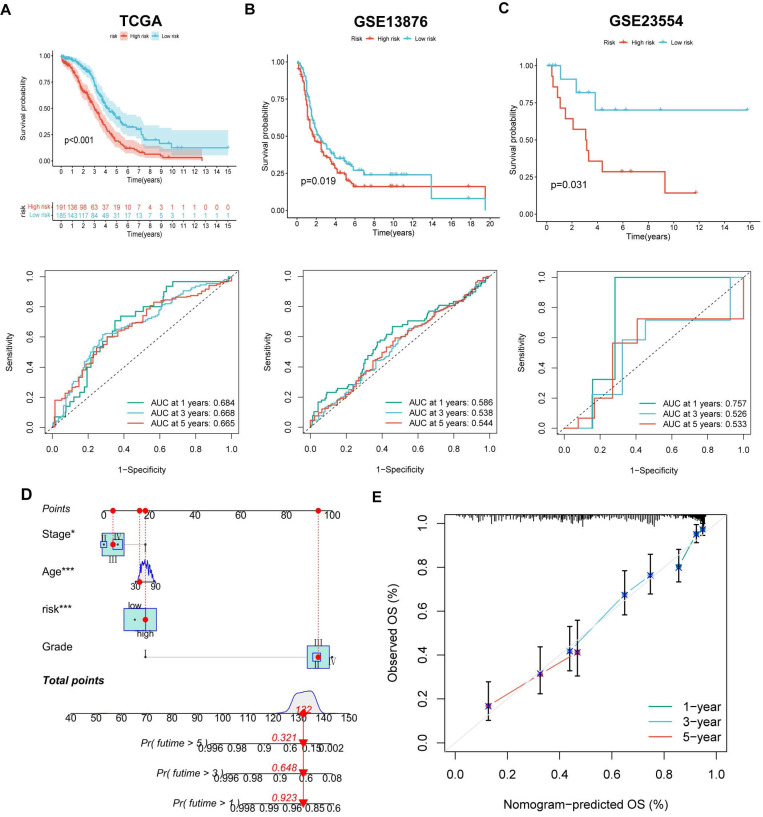
The efficiency of the risk score in predicting patient survival. (A-C) K-M and ROC curves revealed the prognostic values among training and validation cohorts. (D) Constructing a nomogram using the risk score and some risk clinical characteristics. (E) Calibration plot showed the differences among actual survival rates and nomogram-predicted survival rates.

The LASSO and COX regression analysis methods were performed on DEGs to identify four signature genes, ALDH1A2, P2RY14, KIF26A and GDF6 (Figure 5A-B). Meanwhile, the coefficient values of these genes were shown in figure 5C. The risk score was calculated by assessing the expression levels of four signature genes and their corresponding coefficient values, as indicated by the following formula: risk score = [expressed level of ALDH1A2 × (0.169)] + [expressed level of P2RY14 × (-0.525)] + [expressed level of KIF26A × (0.281)] + [expressed level of GDF6 × (0.262)].The Sankey diagram illustrates that OV samples can be categorized into two distinct MRGs molecular clusters, two gene clusters, and two risk groups based on their respective risk scores (Figure 5D). The risk scores differed between the two molecular clusters and two gene clusters (Figure 5E-F). There were significant differences in the 51 prognostic-related MRGs between the high- and low-risk groups (Figure 5G). A heatmap demonstrated the risk coefficient values of the four signature genes (Figure 5H). Subsequently, we found there was a higher mortality rate among high-risk OV patients (Figure 5I). Risk factors were analyzed in the dataset by univariate and multivariate Cox analyses (Figure 5J-K). Additionally, KM and ROC analyses on one experimental cohort were performed to further explore the efficiency of the risk score in predicting patient survival(Figure 6A, TCGA dataset, p < 0.001, 1-year AUC = 0.684, 3-year AUC = 0.668, 5-year AUC = 0.665) and two validation cohorts (Figure 6B&C, GSE13876, p = 0.019, 1-year AUC = 0.586, 3-year AUC = 0.538, 5-year AUC = 0.544 and GSE26193, p = 0.356, 1-year AUC = 0.362, 3-year AUC = 0.542, 5-year AUC = 0.503). Then we conducted a nomogram based on four risk factors, grade, stage, age and risk scores, from the results of single and multiple factor cox analysis (Figure 6D). The calibration plot demonstrated a close agreement between the nomogram-predicted survival rates and the actual survival rates (Figure 6E).

### The immune cell infiltration and TME in high- and low-risk groups

**Figure 7:**
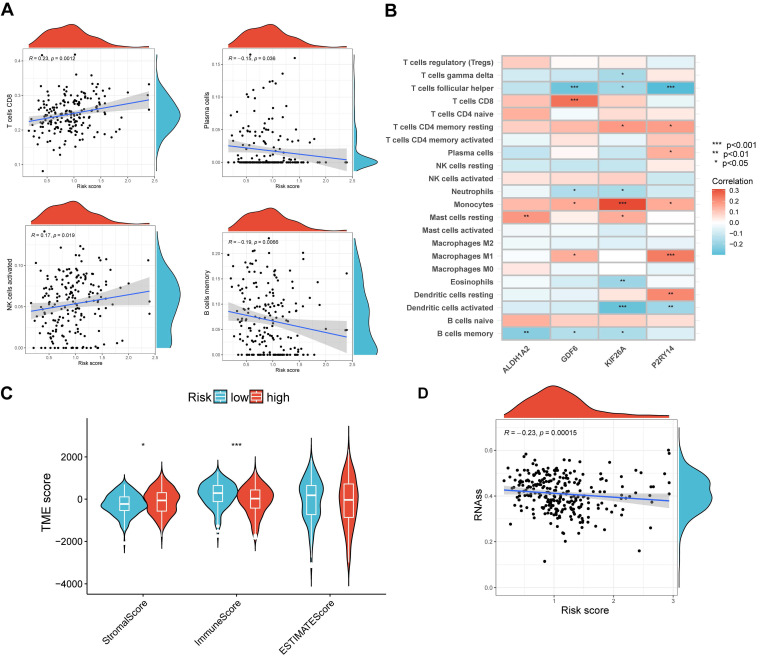
Evaluation of TME in different risk scores. (A) Correlation among different immune cell types and risk score. (B) Relation among four signature genes and the abundance of immune cells. (C) Connection among TME-related scores and risk score. (D) Correlation between risk scores and RNAss. *p < 0.05; ***p < 0.001.

In order to validate the precision of our risk score in making predictions, an examination was conducted on the immune condition of both the high- and low-risk groups. The analysis revealed a correlation between the risk scores and four distinct types of immune cells, namely CD8 cells, plasma cells, activated NK cells, and memory B cells (Figure 7A). Additionally, a significant association was observed between the four signature genes and numerous infiltrations of immune cells, including follicular helper T cells (Figure 7B). Figure 7C demonstrated that the low-risk group exhibited a significantly lower stromal score (p < 0.05) and a significantly higher immune score (p < 0.001) compared to the high-risk group. Additionally, our findings revealed a strong correlation between high RNA stemness scores (RNAss) and lower risk scores (Figure 7D). These results provide evidence that the signature genes we identified are intricately linked to the immune profile of the cells.

### Relationship between risk score and IC50 values of therapeutic drugs

**Figure 8:**
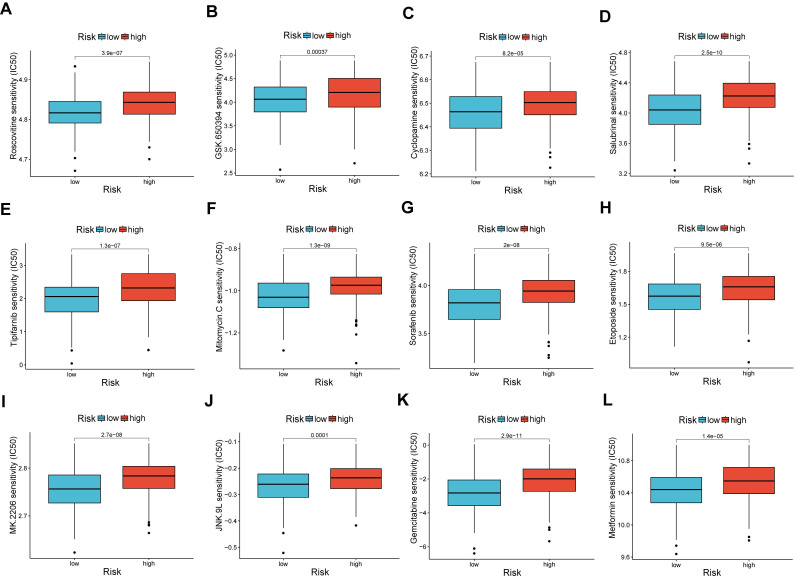
Analysis of drug susceptibility. (A-L) The significant differences in IC50 of therapeutic drugs in low- and high-risk groups. *p < 0.05; **p < 0.01; and ***p < 0.001.

A comparison of chemotherapy drug sensitivity between high-risk and low-risk OV patients revealed that the IC50 values for roscovitine, cyclopamine, and other drugs were higher in the high-risk group. This suggests a correlation between risk scores and drug sensitivity (Figure 8A-L).

### Analysis of Single cell RNA-sequencing for four signature genes

**Figure 9:**
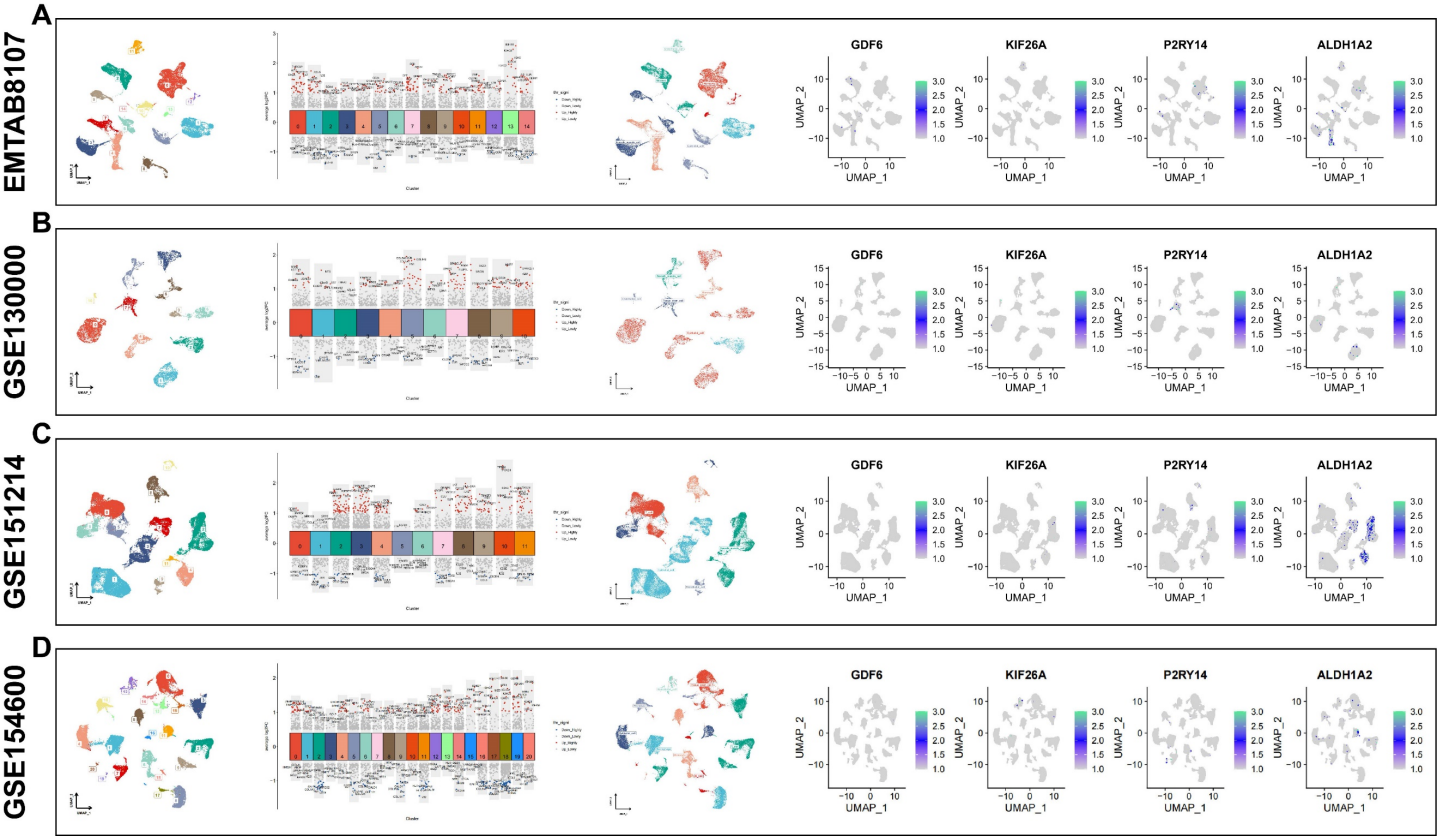
Analysis of Single-cell RNA sequencing. Using four single-cell datasets EMTAB8107(A), GSE130000 (B), GSE151214 (C) and GSE154600 (D) to verify the four signature genes expression location in various cell types. UMAP is used to visualize the dimension reduction clustering of the dataset, while the violin map is employed to display characteristic genes and cell annotation.

Through analyzing four single-cell datasets, EMTAB8107, GSE130000, GSE151214, GSE154600, we found GDF6 was intensely expressed in fibroblast cells, KIF26A was intensely expressed in endothelial cells and smooth muscle cells, P2RY14 was intensely expressed in monocytes, and ALDH1A2 was intensely expressed in smooth muscle cells (Figure 9A-D).

### The expression levels of the four signature genes in OV and normal healthy controls

**Figure 10:**
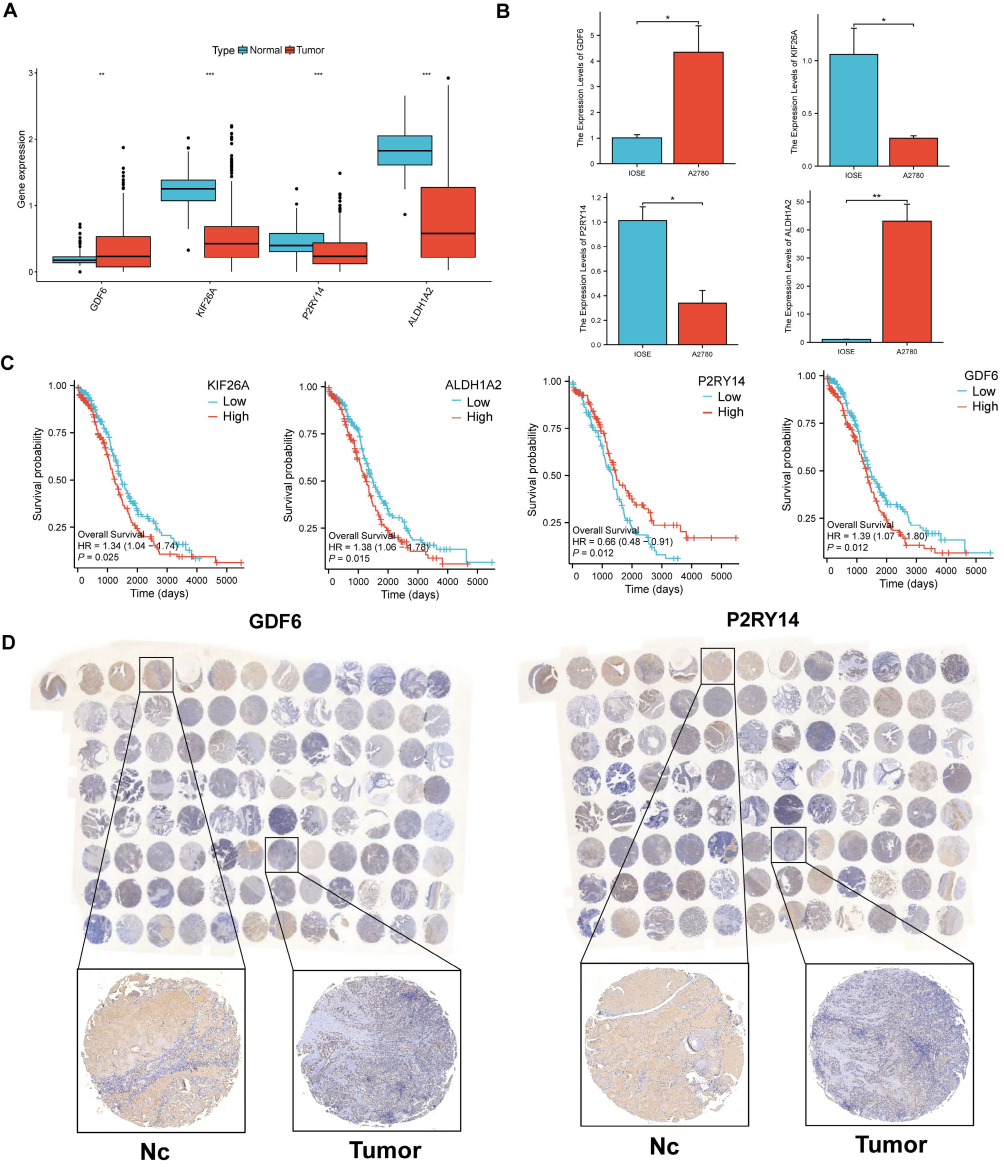
The comparison of levels of signature genes expression in tumor and normal controls. (A) Differences of expression levels of the four signature genes among the normal ovarian tissues and Ovarian cancer tissues (TCGA and GTEx database). (B) The relative mRNA levels of the four signature genes between normal ovarian cell lines and OV cell lines. (C) The prognostic value of a 4 sigurenaure gene for patients in TCGA has been confirmed through Kaplan-Meier analysis. (D) The immunohistochemical staining of tissue microarray shows 4 signature genes expressions on protein level between OV and normal control tissues.

To investigate the expressional condition of the four signature genes, we firstly compared the expression levels of these genes in the OV patients and healthy controls according to mRNA expression profile of TCGA database. The results showed GDF6 had higher expression in OV patients, while KIF26A, P2RY14 and ALDH1A2 were higher expression in normal controls (Figure 10A). Then we discovered GDF6 was also highly expressed in OV cell lines (A2780), and KIF26A and P2RY14 were highly expressed in normal ovarian cell lines by qRT-PCR (Figure 10B). Combined with the survival curve, we found that low expression of P2RY14 and high expression of GDF6 was associated with poor prognosis of OV (Figure 10C). Moreover, the investigation of protein expression levels of GDF6 and P2RY14 in OV and non-tumor tissues was conducted through the utilization of immune histochemistry (IHC) on the tissue microarray. The findings revealed that both GDF6 and P2RY14 exhibited low expression in OV, as depicted in Figure 10D.

### Exploring the function of P2RY14 with combining in vitro experiments

**Figure 11:**
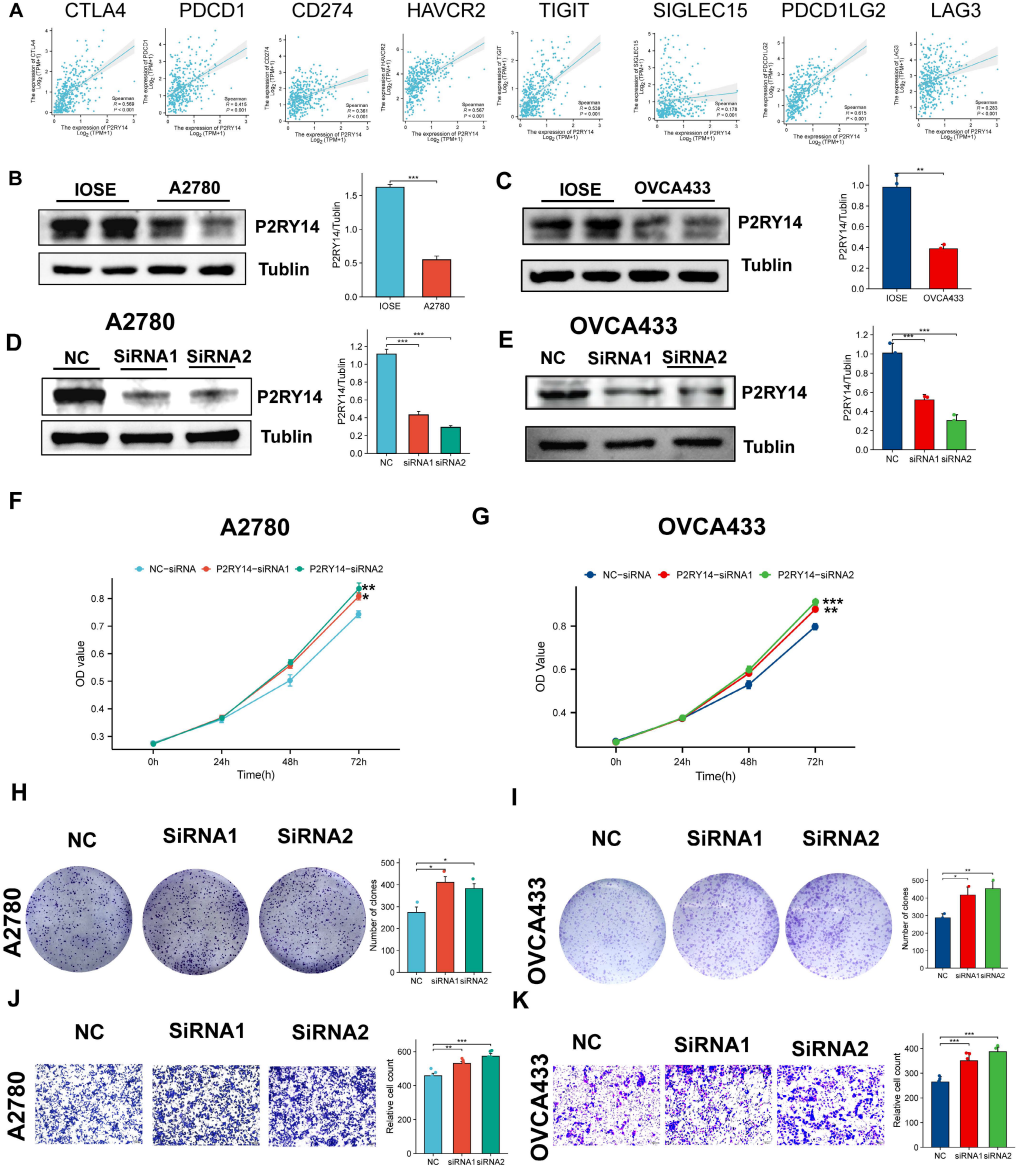
The function of P2RY14. (A) The relationship among the expression levels of P2RY14 and eight immune checkpoint gene in OV. (B-C) Comparison of expression levels of P2RY14 between OV cell lines and normal ovarian cell lines. (D-E) Knocking out the P2RY14 in OV cell lines by two siRNA. Assessing the effects of P2RY14 by CCK8 (F-G) and colony formation (H-I). (J-K) Evaluation of migration by transwell assay.

Based on the above findings, it is speculated that the low expression of P2RY14 may be closely connected to the poor prognosis of OV. To further comprehend the significance of P2RY14 in OV, the relationship between P2RY14 expression and immune checkpoint and biological function were investigated. As shown in Figure 11A, the expression levels of eight immune checkpoint genes including LAG3, SIGLEC15, TIGIT, CD274, HAVCR2, PDCD1, CTLA4 and PDCD1LG2 were all positively linked with P2RY14 in OV (Figure 11A). The results of WB demonstrated the P2RY14 was expressed at lower levels in A2780 and OVCA433 (Figure 11B-C). To further explore the function of P2RY14 in A2780 and OVCA433, P2RY14 protein expression were knocked down by siRNA (Figure 11D-E). CCK8 (Figure 10F-G) and colony formation (Figure 10H-I) assays demonstrated that silencing P2RY14 expression promoted cell proliferation, and Transwell (Figure 11 J-K) assays demonstrated that silencing P2RY14 expression inhibited cell migration in A2780 and OVCA433 cell lines.

## Discussion

Over the past few decades, the treatment of OV has been rapidly updated and developed, including neoadjuvant chemotherapy, immunotherapy, PARP inhibitors, among other approaches. However the 5-year survival rate is still very low, and the prognosis is not optimistic^18^-^20^. Therefore, it is very important to effectively predict the prognosis of OV and explore more sensitive drugs. At present, there have been studies using bioinformatics analysis based on immune-related gene sets to identify some signature genes of OV and construct prognostic signature models to assess patient risk^21^, ^22^. However, current research is still unable to predict and assess the risk of OV patients with great accuracy. This study constructed a prognostic signature model of OV using prognosis-related MRGs that have been empirically indicated to be significantly associated with tumor development. This model is anticipated to offer additional insights for the assessment of disease risk and the tailoring of personalized treatment approaches.

Relative to normal cells, tumor cells typically reprogram metabolism pathways to meet the growth demands of malignant cells^23^. A common pattern of metabolism pathways dysregulation in cancer is mutations in MRGs involved in glycolysis, fatty acid synthesis, serine metabolism, and so on^24^. Our study revealed that many prognostic-related MRGs are mutated in patients with OV, suggesting a possible association between MRGs and the development and progression of OV. Subsequently, the OV individuals were classified into two molecular clusters according to the expression levels of the aforementioned MRGs. Our investigation unveiled significant disparities in terms of survival outcomes, expression of immune checkpoint genes and infiltration of immune cells, between the two molecular clusters, thus validating the credibility of the clustering methodology employed utilizing the MRGs. Consequently, we identified four prognostic signature genes, namely GDF6, KIF26A, P2RY14, and ALDH1A2, from the DEGs observed within the two molecular clusters, thereby contributing to the understanding of OV. Low expression of P2RY14 and ALDH1A2 has been demonstrated to be related to poor prognosis of OV 25, 26. Furthermore, previous studies have proved the inhibitory effects of ALDH1A2 on the migratory and proliferative capabilities of ovarian cancer cells^26^. Once the signature genes and their corresponding risk coefficients have been identified, it becomes possible to calculate a risk score for each sample, thereby facilitating the categorization of ovarian cancer patients into high- and low-risk groups based on their individual risk scores. Additionally, to enhance the accuracy of prognostic predictions, a nomogram incorporating clinical features such as tumor grade, age, and stage has been developed.

Recently, immunotherapy has been proved to improve survival in a variety of cancers such as renal cell carcinoma, lung cancer, and metastatic melanoma^7^. Given that this is an evolving field, and because PARP inhibitors can work against immune checkpoints PD-L1 and CTLA-4, immunotherapy is a potential novel frontier for the treatment of recurrent OV^27^, ^28^. After focusing on the characteristics of the tumor for a long time, the scientists found that the behavior of the non-tumor cells became as meaningful as the tumor itself^29^. The presence of TME and immune cell infiltrations in tumors has been demonstrated to be significant markers for assessing the efficacy of immunotherapy^30^. TME encompasses cancer cells surrounded by a diverse array of non-malignant cell types and vascularized extracellular matrix^31^. The ESTIMATE algorithm was employed to compute the relative abundance of immune-stromal components within TME, which were further categorized into immune score, stromal score, and ESTIMATE score. Our analysis showed that the low-risk group exhibited elevated immune score and ESTIMATE score, potentially indicating heightened infiltration of immune cells. Notably, OV individuals with lower risk scores displayed improved prognosis and a more favorable TME. In order to corroborate our findings, we conducted an analysis of drug susceptibility within the two identified risk groups. Our investigation revealed that the low-risk group showed significantly lower IC50 values in relation to 15 distinct therapeutic drugs. This observation implies that individuals classified as low-risk may possess heightened sensitivity towards chemotherapy.

Among the various techniques for evaluating gene expression, scRNA-seq is a relatively new technique that measures the level of gene expression in each respective transcript within each cell in a sample and can represent the distribution of this expression across each cell subpopulation^32^, ^33^. The intensive expression of KIF26A and ALDH1A2 on smooth muscle cells suggests their potential involvement in tumor progression. Furthermore, significant differences in the expression levels of these four characteristic genes were observed between ovarian cancer patients and healthy controls.

To provide additional evidence for the predictive effectiveness of the signature genes, several in vitro experiments were performed to compare the expression levels of these genes. The results obtained from qRT-PCR analysis demonstrated distinct expression levels of the four genes in OV cells in comparison to normal ovarian cells, which were basically consistent with the statistical results derived from clinical samples obtained from a publicly accessible database. Furthermore, we validated through IHC experiments that the expression of P2RY14 and GDF6 was decreased in tumor tissues. As a member of the original family of extracellular nucleotide-sensitive receptors^34^, UDP-glucose-specific G(i) protein-coupled P2Y receptor (P2RY14) has been extensively demonstrated to play a role in the development and progression of various malignancies, including glioma cells^35^, lung adenocarcinoma^36^, acute lymphoblastic leukemia^37^. Therefore, we explore the function of P2RY14 by combining in with vitro experiments, our investigation demonstrated that the knockout of P2RY14 resulted in enhanced proliferation capacity and migration of OV cells. Moreover, the efficacy of suitable immune checkpoint modulation in immunotherapy has been widely acknowledged^38^. Intriguingly, our results indicate a significant correlation between P2RY14 and eight distinct immune checkpoints, thereby reinforcing the potential of P2RY14 targeting to enhance the therapeutic outcomes of immunotherapy. And for the first time, the function of P2RY14 in OV was explored in this study. The findings of our study indicate a potential correlation between low expression of P2RY14 and unfavorable prognostic outcomes in ovarian cancer. P2RY14 may act as a tumor suppressor gene in ovarian cancer, and further experiments are needed to ascertain its definitive role.

However, our research still exhibits certain limitations. Despite the utilization of the TCGA public database, which currently boasts the largest sample size among ovarian cancer patients for the identification of signature genes, further enhancement of statistical effectiveness necessitates a larger sample size. Secondly, we found that knocking down P2RY14 can promote the growth and migration of OV cells. Nevertheless, the underlying mechanism by which P2RY14 regulates tumor growth remains unclear.

## Conclusions

In conclusion, our study has successfully constructed a prognostic signature model that demonstrates significant efficacy in predicting the prognosis of ovarian cancer patients. Furthermore, we have successfully identified and validated four signature genes. Additionally, our findings suggest that downregulation expression of P2RY14 facilitates the proliferation and migration of ovarian cancer cells, potentially contributing to the unfavorable prognosis associated with this disease.

## Supplementary Material

Supplementary tables.

## References

[1] Siegel RL, Miller KD, Jemal A (2020). Cancer statistics, 2020. CA: a cancer journal for clinicians.

[2] Kuchenbaecker KB, Hopper JL, Barnes DR, Phillips KA, Mooij TM, Roos-Blom MJ (2017). Risks of Breast, Ovarian, and Contralateral Breast Cancer for BRCA1 and BRCA2 Mutation Carriers. Jama.

[3] Norquist BM, Harrell MI, Brady MF, Walsh T, Lee MK, Gulsuner S (2016). Inherited Mutations in Women With Ovarian Carcinoma. JAMA oncology.

[4] Kuroki L, Guntupalli SR (2020). Treatment of epithelial ovarian cancer. BMJ (Clinical research ed).

[5] Cho KR, Shih Ie M (2009). Ovarian cancer. Annual review of pathology.

[6] Wright AA, Bohlke K, Armstrong DK, Bookman MA, Cliby WA, Coleman RL (2016). Neoadjuvant chemotherapy for newly diagnosed, advanced ovarian cancer: Society of Gynecologic Oncology and American Society of Clinical Oncology Clinical Practice Guideline. Gynecol Oncol.

[7] Robert C, Ribas A, Schachter J, Arance A, Grob JJ, Mortier L (2019). Pembrolizumab versus ipilimumab in advanced melanoma (KEYNOTE-006): post-hoc 5-year results from an open-label, multicentre, randomised, controlled, phase 3 study. The Lancet Oncology.

[8] Wang X, Xu Y, Dai L, Yu Z, Wang M, Chan S (2022). A novel oxidative stress- and ferroptosis-related gene prognostic signature for distinguishing cold and hot tumors in colorectal cancer. Front Immunol.

[9] Chen J, Wang D, Chan S, Yang Q, Wang C, Wang X (2023). Development and validation of a novel T cell proliferation-related prognostic model for predicting survival and immunotherapy benefits in melanoma. Aging.

[10] Martínez-Reyes I, Chandel NS (2021). Cancer metabolism: looking forward. Nature reviews Cancer.

[11] Huang D, Li C, Zhang H (2014). Hypoxia and cancer cell metabolism. Acta Biochim Biophys Sin (Shanghai).

[12] Bian X, Liu R, Meng Y, Xing D, Xu D, Lu Z (2021). Lipid metabolism and cancer. The Journal of experimental medicine.

[13] Tabe Y, Lorenzi PL, Konopleva M (2019). Amino acid metabolism in hematologic malignancies and the era of targeted therapy. Blood.

[14] Zhang H, Liu Y, Hu D, Liu S (2022). Identification of Novel Molecular Therapeutic Targets and Their Potential Prognostic Biomarkers Based on Cytolytic Activity in Skin Cutaneous Melanoma. Frontiers in oncology.

[15] Liu Y, Zhang H, Mao Y, Shi Y, Wang X, Shi S (2023). Bulk and single-cell RNA-sequencing analyses along with abundant machine learning methods identify a novel monocyte signature in SKCM. Front Immunol.

[16] Wang X, Sun R, Chan S, Meng L, Xu Y, Zuo X (2022). PANoptosis-based molecular clustering and prognostic signature predicts patient survival and immune landscape in colon cancer. Front Genet.

[17] Chen Y (2022). Identification and Validation of Cuproptosis-Related Prognostic Signature and Associated Regulatory Axis in Uterine Corpus Endometrial Carcinoma. Front Genet.

[18] Wright AA, Bohlke K, Armstrong DK, Bookman MA, Cliby WA, Coleman RL (2016). Neoadjuvant Chemotherapy for Newly Diagnosed, Advanced Ovarian Cancer: Society of Gynecologic Oncology and American Society of Clinical Oncology Clinical Practice Guideline. Journal of clinical oncology: official journal of the American Society of Clinical Oncology.

[19] Liu JF, Herold C, Gray KP, Penson RT, Horowitz N, Konstantinopoulos PA (2019). Assessment of Combined Nivolumab and Bevacizumab in Relapsed Ovarian Cancer: A Phase 2 Clinical Trial. JAMA oncology.

[20] Pujade-Lauraine E, Ledermann JA, Selle F, Gebski V, Penson RT, Oza AM (2017). Olaparib tablets as maintenance therapy in patients with platinum-sensitive, relapsed ovarian cancer and a BRCA1/2 mutation (SOLO2/ENGOT-Ov21): a double-blind, randomised, placebo-controlled, phase 3 trial. The Lancet Oncology.

[21] Feng S, Xu Y, Dai Z, Yin H, Zhang K, Shen Y (2022). Integrative Analysis From Multicenter Studies Identifies a WGCNA-Derived Cancer-Associated Fibroblast Signature for Ovarian Cancer. Front Immunol.

[22] Shen S, Wang G, Zhang R, Zhao Y, Yu H, Wei Y (2019). Development and validation of an immune gene-set based Prognostic signature in ovarian cancer. EBioMedicine.

[23] DeBerardinis RJ, Chandel NS (2016). Fundamentals of cancer metabolism. Science advances.

[24] Stine ZE, Walton ZE, Altman BJ, Hsieh AL, Dang CV (2015). MYC, Metabolism, and Cancer. Cancer discovery.

[25] Shu C, Liu L, Chen X, Xue J, Fei J, Wang J (2023). ncRNA-mediated low expression of P2RY14 correlates with poor prognosis and tumor immune infiltration in ovarian carcinoma. Ann Transl Med.

[26] Wang Y, Shao F, Chen L (2018). ALDH1A2 suppresses epithelial ovarian cancer cell proliferation and migration by downregulating STAT3. OncoTargets and therapy.

[27] Gandhi L, Rodríguez-Abreu D, Gadgeel S, Esteban E, Felip E, De Angelis F (2018). Pembrolizumab plus Chemotherapy in Metastatic Non-Small-Cell Lung Cancer. The New England journal of medicine.

[28] Konstantinopoulos PA, Waggoner S, Vidal GA, Mita M, Moroney JW, Holloway R (2019). Single-Arm Phases 1 and 2 Trial of Niraparib in Combination With Pembrolizumab in Patients With Recurrent Platinum-Resistant Ovarian Carcinoma. JAMA oncology.

[29] Fridman WH, Zitvogel L, Sautès-Fridman C, Kroemer G (2017). The immune contexture in cancer prognosis and treatment. Nature reviews Clinical oncology.

[30] Fridman WH, Pagès F, Sautès-Fridman C, Galon J (2012). The immune contexture in human tumours: impact on clinical outcome. Nature reviews Cancer.

[31] Chen DS, Mellman I (2017). Elements of cancer immunity and the cancer-immune set point. Nature.

[32] Tang F, Barbacioru C, Wang Y, Nordman E, Lee C, Xu N (2009). mRNA-Seq whole-transcriptome analysis of a single cell. Nature methods.

[33] Lähnemann D, Köster J, Szczurek E, McCarthy DJ, Hicks SC, Robinson MD (2020). Eleven grand challenges in single-cell data science. Genome Biol.

[34] Abbracchio MP, Burnstock G, Verkhratsky A, Zimmermann H (2009). Purinergic signalling in the nervous system: an overview. Trends in neurosciences.

[35] Curet MA, Watters JJ (2018). P2Y14 receptor activation decreases interleukin-6 production and glioma GL261 cell proliferation in microglial transwell cultures. Journal of neuro-oncology.

[36] Xu T, Xu S, Yao Y, Chen X, Zhang Q, Zhao X (2022). P2RY14 downregulation in lung adenocarcinoma: a potential therapeutic target associated with immune infiltration. Journal of thoracic disease.

[37] Shah K, Moharram SA, Kazi JU (2018). Acute leukemia cells resistant to PI3K/mTOR inhibition display upregulation of P2RY14 expression. Clin Epigenetics.

[38] Chae YK, Arya A, Iams W, Cruz MR, Chandra S, Choi J (2018). Current landscape and future of dual anti-CTLA4 and PD-1/PD-L1 blockade immunotherapy in cancer; lessons learned from clinical trials with melanoma and non-small cell lung cancer (NSCLC). Journal for immunotherapy of cancer.

